# Multi-Omics Analysis of Microglial Extracellular Vesicles From Human Alzheimer’s Disease Brain Tissue Reveals Disease-Associated Signatures

**DOI:** 10.3389/fphar.2021.766082

**Published:** 2021-12-02

**Authors:** Whitaker Cohn, Mikhail Melnik, Calvin Huang, Bruce Teter, Sujyoti Chandra, Chunni Zhu, Laura Beth McIntire, Varghese John, Karen H. Gylys, Tina Bilousova

**Affiliations:** ^1^ Drug Discovery Lab, Department of Neurology, University of California, Los Angeles, Los Angeles, CA, United States; ^2^ School of Nursing, University of California, Los Angeles, Los Angeles, CA, United States; ^3^ Taub Institute for Research on Alzheimer’s Disease and the Aging Brain, Department of Pathology and Cell Biology, Columbia University Medical Center, New York, NY, United States

**Keywords:** Alzheimer’s disease, extracellular vesicles, exosomes, microglia, omics analysis

## Abstract

Alzheimer’s disease (AD) is the most common cause of dementia, yet there is no cure or diagnostics available prior to the onset of clinical symptoms. Extracellular vesicles (EVs) are lipid bilayer-delimited particles that are released from almost all types of cell. Genome-wide association studies have linked multiple AD genetic risk factors to microglia-specific pathways. It is plausible that microglia-derived EVs may play a role in the progression of AD by contributing to the dissemination of insoluble pathogenic proteins, such as tau and Aβ. Despite the potential utility of EVs as a diagnostic tool, our knowledge of human brain EV subpopulations is limited. Here we present a method for isolating microglial CD11b-positive small EVs from cryopreserved human brain tissue, as well as an integrated multiomics analysis of microglial EVs enriched from the parietal cortex of four late-stage AD (Braak V-VI) and three age-matched normal/low pathology (NL) cases. This integrated analysis revealed 1,000 proteins, 594 lipids, and 105 miRNAs using shotgun proteomics, targeted lipidomics, and NanoString nCounter technology, respectively. The results showed a significant reduction in the abundance of homeostatic microglia markers P2RY12 and TMEM119, and increased levels of disease-associated microglia markers FTH1 and TREM2, in CD11b-positive EVs from AD brain compared to NL cases. Tau abundance was significantly higher in AD brain-derived microglial EVs. These changes were accompanied by the upregulation of synaptic and neuron-specific proteins in the AD group. Levels of free cholesterol were elevated in microglial EVs from the AD brain. Lipidomic analysis also revealed a proinflammatory lipid profile, endolysosomal dysfunction, and a significant AD-associated decrease in levels of docosahexaenoic acid (DHA)-containing polyunsaturated lipids, suggesting a potential defect in acyl-chain remodeling. Additionally, four miRNAs associated with immune and cellular senescence signaling pathways were significantly upregulated in the AD group. Our data suggest that loss of the homeostatic microglia signature in late AD stages may be accompanied by endolysosomal impairment and the release of undigested neuronal and myelin debris, including tau, through extracellular vesicles. We suggest that the analysis of microglia-derived EVs has merit for identifying novel EV-associated biomarkers and providing a framework for future larger-scale multiomics studies on patient-derived cell-type-specific EVs.

## Introduction

Alzheimer’s disease (AD) is the most common cause of dementia, yet there is no cure or diagnostics available prior to the onset of clinical symptoms (2021 Alzheimer’s disease facts and figures, 2021). The main neuropathological hallmarks of AD include the accumulation of amyloid beta peptide (Aβ)-containing plaques, hyperphosphorylated tau protein (p-tau)-composed neurofibrillary tangles, extensive neuroinflammation, synaptic loss, and neuronal cell death (Serrano-Pozoe et al., 2011). Numerous anti-AD drug candidates have proven to be effective in AD animal models but subsequently failed in clinical trials (Mehta et al., 2017). These failures can be attributed to three factors: 1) limited knowledge of the complex cellular and molecular mechanisms causing disease onset; 2) late initiation of the experimental treatments; and 3) inadequate monitoring of the treatment effects due to the absence of a biomarker panel that provides accurate longitudinal information regarding disease progression (Yiannopoulou et al., 2019).

Small extracellular vesicles (EVs), originating either from the plasma membrane (microvesicles) or from multivesicular bodies (exosomes), play a role in many of the major pathological and physiological pathways altered in AD, including Aβ aggregation (Dinkins et al., 2014; Kokubo, et al., 2005; Rajendran, et al., 2006; Yuyama, et al., 2014), spread of tau and Aβ seeds (Asai, et al., 2015; Bilousova, et al., 2018; Bilousova, et al., 2020; Joshi, et al., 2014; Sardar Sinha, et al., 2018), neuroinflammation (Pascual et al., 2020), synaptic transmission (An, et al., 2013), cell death (G. Wang, et al., 2012; Winston, et al., 2019), and senescence (D'Anca, et al., 2019). Interestingly, the two greatest genetic risk factors for late-onset AD (LOAD), apolipoprotein E (apoE) and bridging integrator-1(Bin1), are involved in EV biogenesis and/or cargo sorting (Crotti, et al., 2019; Peng, et al., 2019), suggesting direct involvement of EVs in the development AD pathophysiology. Moreover, the molecular composition of EVs reflects the state and makeup of their cells of origin, and thus, they may be an invaluable resource for identifying important biomarkers of the disease. Indeed, brain-derived EVs cross the blood–brain barrier (BBB) harboring disease-associated molecules such as p-tau and Aβ. In this context, they provide an accessible reservoir of biomarkers that might predict the development of AD at the asymptomatic stage, as well as the conversion from mild cognitive impairment (MCI)/prodromal stage to clinical AD (Fiandaca, et al., 2015; Goetzl et al., 2016; Goetzl et al., 2018; Hornung et al., 2020; Winston, et al., 2016).

Genome-wide association studies (GWAS) have identified multiple AD risk loci located in or near genes preferentially expressed in microglia, suggesting that microglial dysfunction may have a causative role in disease development (Takatori et al., 2019). Moreover, single-cell and single-nuclei RNA sequencing analysis (RNA-seq) revealed the presence of disease-associated microglia (DAM) clusters near Aβ plaques in both animal models and in the human AD brain (Butovsky and Weiner, 2018; Del-Aguila, et al., 2019; Mathys, et al., 2019). DAM populations are characterized by the loss of a homeostatic transcriptional signature, including decreases in purinergic 2Y receptor 12 (P2RY12), P2RY13, transmembrane protein 119 (TMEM119), CX3C chemokine receptor 1 (CX3CR1), and others, and activation of genes responsible for either proinflammatory microglia activation (Stage 1 DAM) or a neurodegeneration restrictive phenotype (Stage 2 DAM). Upregulation of apoE, ferritin heavy chain-1 (FTH-1), beta-2-microglobulin (B2m), major histocompatibility complex class I (MHC class 1), and DAP12 are changes associated with the Stage 1 DAM transcriptome profile, while increases in the triggering receptor expressed on myeloid cells 2 **(**TREM 2) and exosomal markers CD63 and CD9 are part of the Stage 2 DAM program (Deczkowska, et al., 2018; Xue and Du, 2021). Upregulation of exosomal markers suggests that an increase in exosome production may help to resolve inflammation; on the other hand, microglial EVs, specifically the neutral sphingomyelinase 2 (nSMase2)-dependent exosomal population, take part in spreading tau pathology *in vitro* and in mouse models of tauopathy (Asai, et al., 2015; Maphis, et al., 2015).

Despite progress identifying cell-specific EVs in body fluids (Fiandaca, et al., 2015; Goetzl et al., 2016; Goetzl, Mustapic, et al., 2016; Wang, et al., 2012; Willis, et al., 2017), it is important to note that little is known about the composition of cell-type-specific EV subpopulations isolated from human brain tissue. Changes in the brain EV proteome in human AD cases and animal models suggest the enrichment of a neurodegenerative microglial signature in bulk brain EV preparations (Muraoka, et al., 2020; Muraoka, et al., 2021). Our knowledge of the miRNA transcriptome and lipidome of AD brain-derived EVs is even more limited (Cheng, et al., 2020; Su, et al., 2021). Here we specifically focus on human microglia-derived EVs isolated from cryopreserved human tissue from late AD (Braak V-VI) and normal/low pathology (NL) cases in order to characterize their molecular profiles utilizing an integrative approach combining proteomic, transcriptomic (miRNA), and lipidomic analyses.

## Materials and Methods

### Purification of Small Microglial EVs From Cryopreserved Human Brain Tissue

Human brain parietal cortex samples were prepared as described (Gylys and Bilousova, 2017) using tissue from cases with postmortem interval (PMI) less than or equal to 7 h. Briefly, following harvesting of the human brain from Brodmann area A7, A39, or A40, the tissue was finely minced (1–3 mm fragments) on ice and suspended in a solution of sucrose and protease inhibitors (0.32 M sucrose, 2 mM EDTA, 2 mM EGTA, 0.2 mM PMSF, 1 mM NaPP, 5 mM NaF, 10 mM Tris-HCL, pH 8.0), with 10 ml sucrose buffer/g tissue, and then slowly frozen to –80°C; this protocol is based on a described method (Dodd, et al., 1986). On the day of EV isolation, the cryopreserved brain tissue was quickly thawed at 37°C, centrifuged (1,000x*g*, 2 min, 4°C) to remove cryopreservation buffer, and weighed. The tissue was gently dissociated using an adult brain dissociation kit and a GentleMACS dissociator (Miltenyi Biotec) according to the instructions of the manufacturer (0.5 g of tissue per enzymatic reaction). In a recent study comparing six different enzyme protocols for cell dissociation from the rodent brain, this kit yielded the highest number of live cells (Hussain, et al., 2018). After the dissociation step, the cell suspension was passed through a MACS Smart strainer (70 μm; Miltenyi Biotec). Small EV fractions were purified by sequential centrifugation steps including sucrose density gradient ultracentrifugation followed by washing steps as described (Vella, et al., 2017). This protocol for brain EV isolation has been extensively validated in our previous publications and by others, and enrichment of small EVs in the 0.6 M sucrose layer (F2) has been demonstrated (Bilousova, et al., 2020; Huang, et al., 2020; Vassileff, et al., 2020; Zhu, et al., 2021). Final F2 pellets were resuspended in 25 mM trehalose in PBS, pH 7.4 with a protease and phosphatase inhibitor cocktail (ThermoFisher); the volume of the resuspension solution was adjusted based on the original brain tissue weight (1 g of tissue/150 µl solution). Trehalose prevents EV aggregation and serves as a cryopreservant (Bosch, et al., 2016). Small portions of the F2 small EV fractions were frozen at −80°C for further characterization (see below), and the remainder was used for immunoprecipitation (IP) of microglial EVs using antibodies for the myeloid cell-specific marker CD11b (BioLegend; Clone M1/70) or for control IP reactions with isotype control rat IgG2b antibodies (BioLegend; Clone RTK4530). Covalent coupling of antibodies to Dynabeads M-270 epoxy beads was performed with the Dynabead antibody coupling kit (ThermoFisher; 14311D) according to the instructions of the manufacturer. F2 fractions were incubated with a human-specific FcR blocking reagent (Milteny biotec) for 5 min on ice (5 µl of the blocking reagent per 100 µl of F2), followed by 1:20 dilution with 1% bovine serum albumin (BSA) in PBS, pH 7.4. We used 1.25 mg of antibody-coupled Dynabeads per 2 ml of the 1:20 diluted F2 sample. IP reactions were incubated overnight at 4°C with rotation, followed by 4 consecutive 10-min washes with rotation: the first wash with 0.1% BSA/PBS, the second wash with 25 mM citrate-phosphate buffer, pH 5 to reduce the amount of nonspecifically bound material as previously described (Heinzelman et al., 2016), followed by two washes with PBS, pH 7.4. Dynabeads with bound microglial EVs were aliquoted based on the original tissue weight (microglial EVs from 1 g of tissue per aliquot) and frozen at -80°C for downstream analysis.

### Transmission Electron Microscopy (TEM)

For quality control purposes, small amounts of purified brain-derived EVs were loaded on the formvar/carbon 400 mesh copper EM grids (Ted Pella, Inc.), incubated for 30 min, then fixed in 2% paraformaldehyde solution (3 min), followed by washes with distilled water, and staining with 2% uranyl acetate solution (3 min). The grids were dried for at least 1 h at room temperature, and the negative-stained EVs were imaged on a JEOL 100CX electron microscope at 60 kV and ×29,000 magnification.

### Tunable Resistive Pulse Sensing Analysis

Size distribution and concentrations of EV samples were measured by the Tunable Resistive Pulse Sensing (TRPS) method using the qNano Gold instrument (Izon Science). NP100 nanopore (particle size range: 50–330 nm) and CPC100 calibration particles were used for the analysis. Data analysis was performed using the qNano instrument software.

### Immunoblotting

Microglial EVs were separated from Dynabeads by incubation in a Tris-Glycine SDS sample buffer without reducing agents at 95 C for 5 min after which immunoblot analysis was performed as described above. Proteins from microglial and total small EV fractions were separated by 10–20% Tris-Glycine SDS-PAGE under reducing (50 mM dithiothreitol) or nonreducing (for tetraspanins) conditions, transferred to PVDF membrane, and probed with primary antibodies (Table 1) followed by HRP-conjugated secondary antibodies (Jackson ImmunoResearch). Chemiluminescent signals were obtained with Super Signal West Femto substrate (Thermo Scientific Pierce 34095), detected using the BioSpectrum 600 imaging system, and quantified using the VisionWorks version 6.6A software (UVP; Upland, CA). Data were analyzed by Student’s t-test.

**TABLE 1:** Antibodies used for immunoblot analysis of small EV (F2) fractions and microglial EVs isolated from human parietal cortex.

Target protein	Company	Clone name	Figure number
CD9	ThermoFisher	TS9	1C, E
CD81	ThermoFisher	M38	1C
Syntenin-1	Abcam	EPR8102	1C
Calnexin	Santa Cruz Biotech	AF18	1C
GM130	Cell Signaling	D6B1	1C
CD63	ThermoFisher	TS63	1D
Tau	ThermoFisher	HT7	1E
CD11b	BioLegend	M1/70	1E
TREM2	Abcam	EPR20243	2D
apoE	Abcam	E6D7	2D

### Proteomic Analysis

One aliquot of frozen microglial EVs isolated from 1 g of parietal cortex tissue from each case presented in Table 2 was used for the proteomic analysis. Microglial EV samples were diluted in lysis buffer [200 uL, 12 mM sodium lauroyl sarcosine, 0.5% sodium deoxycholate, 50 mM triethylammonium bicarbonate (TEAB)] and Halt™ Protease and Phosphatase Inhibitor Cocktail (Thermo Scientific, Waltham, MA), and then were treated with tris(2-carboxyethyl) phosphine (10 mM, 30 min, 60°C), alkylated (chloroacetamide 40 mM, 30 min, 25°C in the dark), and digested with Sequencing Grade Modified Trypsin (Promega, Madison, WI; 1 ug, 37°C, overnight). The samples were then desalted on C18 StageTips according to Rappsilber’s protocol (Rappsilber et al., 2007). The collected eluent was then chemically modified using a TMT10plex Isobaric Label Reagent Set (Thermo Fisher Scientific) per the protocol of the manufacturer. The samples were pooled according to the protein content (1 ug of peptide from each sample) and desalted again according to Rappsilber’s protocol (Rappsilber, et al., 2007). The eluants were injected onto a reverse-phase nanobore HPLC column (AcuTech Scientific, C18, 1.8-um particle size, 360 um x 20 cm, 150 um ID), equilibrated in solvent A, and eluted (300 nl/min) with an increasing concentration of solvent B (acetonitrile/water/FA, 98/2/0.1, v/v/v: min/% B; 0/0, 5/3, 18/7, 74/12, 144/24, 153/27, 162/40, 164/80, 174/80, 176/0, 180/0) using a Dionex UltiMate 3,000 RSLCnano System (Thermo Fisher Scientific). The effluent from the column was directed to a nanospray ionization source connected to an Orbitrap Fusion Lumos Tribrid mass spectrometer (Thermo Fisher Scientific) acquiring mass spectra utilizing the Synchronous Precursor Selection (SPS) MS3 method in which isolation and MS3 fragmentation of MS2 fragment ions eliminate isolation interference and dynamic range compression often observed in isobaric tandem mass tag-based proteomics experiments.

**TABLE 2:** Case information for human samples.

Case ID	Age, years	Sex	PMI	Diagnosis	Aβ plaque pathology in parietal cortex	Neurofibrillary degeneration stage (Braak)
NL1	65	F	6	MSA, Striatonigral degeneration	0	I
NL2	100	F	4	Normal (mild Braak changes)	0	III
NL3	91	F	7	Normal, multiple infarctions	0	II
AD1	86	M	6	AD, vascular dementia	Severe	V
AD2	95	F	6	AD, vascular dementia	Severe	VI
AD3	95	M	5	AD, LB in SN&LC, CAA	Moderate	V
AD4	88	M	7	AD, diffuse LB	Severe	VI

F, female; M, male; AD, Alzheimer disease; CAA, cerebral amyloid angiopathy; MSE, multiple system atrophy; SN, substantia nigra; PMI, postmortem interval; LB, Lewy bodies.

For statistical analysis, the raw data were searched against the Uniprot human reviewed protein database using SEQUEST-HT in Proteome Discoverer (Version 2,4, Thermo Scientific), which provided measurements of relative abundance of the identified peptides. Decoy database searching was used to generate high confidence tryptic peptides (FDR < 1%). Tryptic peptides containing amino acid sequences unique to individual proteins were used to identify and provide relative quantification between proteins in each sample. Between-group comparisons were analyzed using the abundance ratio *p*-value (Student’s t-test). Gene set enrichment gene ontology (GO) and pathway analysis was performed by the STRING database (version 11.5), which was used for functional interpretation of the proteomics data and provided *p*-values corrected by the FDR method (Szklarczyk, et al., 2019).

### MicroRNA Transcriptomics

Microglial EV samples isolated from 1 g of parietal cortex tissue from each case presented in Table 2 were used for RNA purification with the SerMir Exosome RNA Column Purification Kit (Systems Bioscience Inc). Purified RNA samples were run on nCounter human microRNA panel Human v3 miRNA according to the instructions of the manufacturer (NanoString). The panel contains 800 pairs of probes specific for a predefined set of biologically relevant miRNAs. which were combined with a series of internal controls to form a Human miRNA Panel CodeSet (NanoString Technologies). Raw counts for miRNA targets were analyzed using the Nanostring nCounter Digital Analyzer software according to the instructions of the manufacture.

Statistical analysis of NanoString nCounter data was performed as described (Brumbaugh et al., 2011). Only miRNAs that were above background in five out of seven samples from two groups (NL and AD) were selected for the analysis, which resulted in normalized counts and a list of differentially expressed microRNAs that significantly differ between NL and AD groups. Using the miRNet software, we then identified top pathways in the Reactome pathway database, which can be controlled by the miRNAs shown to be significantly altered in the AD group compared to NL by Student's t-test; *p*-values were corrected by the FDR method.

### Lipidomic Analysis

We interrogated 34 classes, including free cholesterol (FC), cholesteryl ester (CE), acyl carnitine (AC), monoacylglycerol (MG), diacylglycerol (DG), triacylglycerol (TG), ceramide (Cer), sphingomyelin (SM), monohexosylceramide (MhCer), sulfatides (Sulf), lactosylceramide (LacCer), monosialodihexosylganglioside (GM3), globotriaosylceramide (GB3), phosphatidic acid (PA), phosphatidylcholine (PC), ether phosphatidylcholine (PCe), phosphatidylethanolamine (PE), plasmalogen phosphatidylethanolamine (Pep), phosphatidylserine (PS), phosphatidylinositol (PI), phosphatidylglycerol (PG), bis(monoacylglycerol)phosphate (BMP), acyl phosphatidylglycerol (AcylPG), lysophosphatidylcholine (LPC), ether lysophosphatidylcholine (LPCe), lysophosphatidylethanolamine (LPE), plasmalogen lysophosphatidylethanolamine (LPEp), lysophosphatidylinositol (LPI), lysophosphatidylserine (LPS), N-Acyl phosphatidylethanolamine (NAPE), N-acyl phosphatidylserine (NAPS), and N-acyl serine (NSer), encompassing 593 individual lipid species. Immuno-isolated microglial EV preparations were subjected to modified Bligh–Dyer lipid extraction (Bligh and Dyer, 1959), dried, resuspended, and subjected to liquid-chromatography tandem mass spectrometry (LC-MS/MS) targeted lipidomics using Agilent 1,260 Infinity HPLC integrated to an Agilent 6490A QQQ mass spectrometer controlled by Masshunter v 7.0 (Agilent Technologies, Santa Clara, CA) in positive and negative ion modes as previously described (Chan, et al., 2012; Guan et al., 2007; Hsu et al., 2004). Quantification of lipid species was accomplished using multiple reaction monitoring (MRM) transitions (Chan, et al., 2012; Guan, et al., 2007; Hsu, et al., 2004) under both positive and negative ionization modes in conjunction with referencing of appropriate internal standards: PA 14:0/14:0, PC 14:0/14:0, PE 14:0/14:0, PG 15:0/15:0, PI 17:0/20:4, PS 14:0/14:0, BMP 14:0/14:0, APG 14:0/14:0, LPC 17:0, LPE 14:0, LPI 13:0, Cer d18:1/17:0, SM d18:1/12:0, dhSM d18:0/12:0, GalCer d18:1/12:0, GluCer d18:1/12:0, LacCer d18:1/12:0, D7-cholesterol, CE 17:0, MG 17:0, 4ME 16:0 diether DG, and D5-TG 16:0/18:0/16:0 (Avanti Polar Lipids, Alabaster, AL). Lipid levels for each sample were calculated by summing the total number of moles of all lipid species measured by all three LC-MS methodologies and then normalizing that total to mol%. The final data are presented as mean mol% with error bars showing mean ± S.E. Graph-pad Prism was used to analyze the lipidomics data.

For statistical analysis, two-way repeated measures (mixed model) ANOVA was completed on individual lipid classes among the lipid species using an unweighted means analysis due to the different n for LN and AD groups. Bonferroni post-test was used to compare replicate means by row (lipid species) in which microglia NL and microglia AD were compared.

## Results

### Microglia-Derived Small EV Isolation and Characterization

**FIGURE 1:**
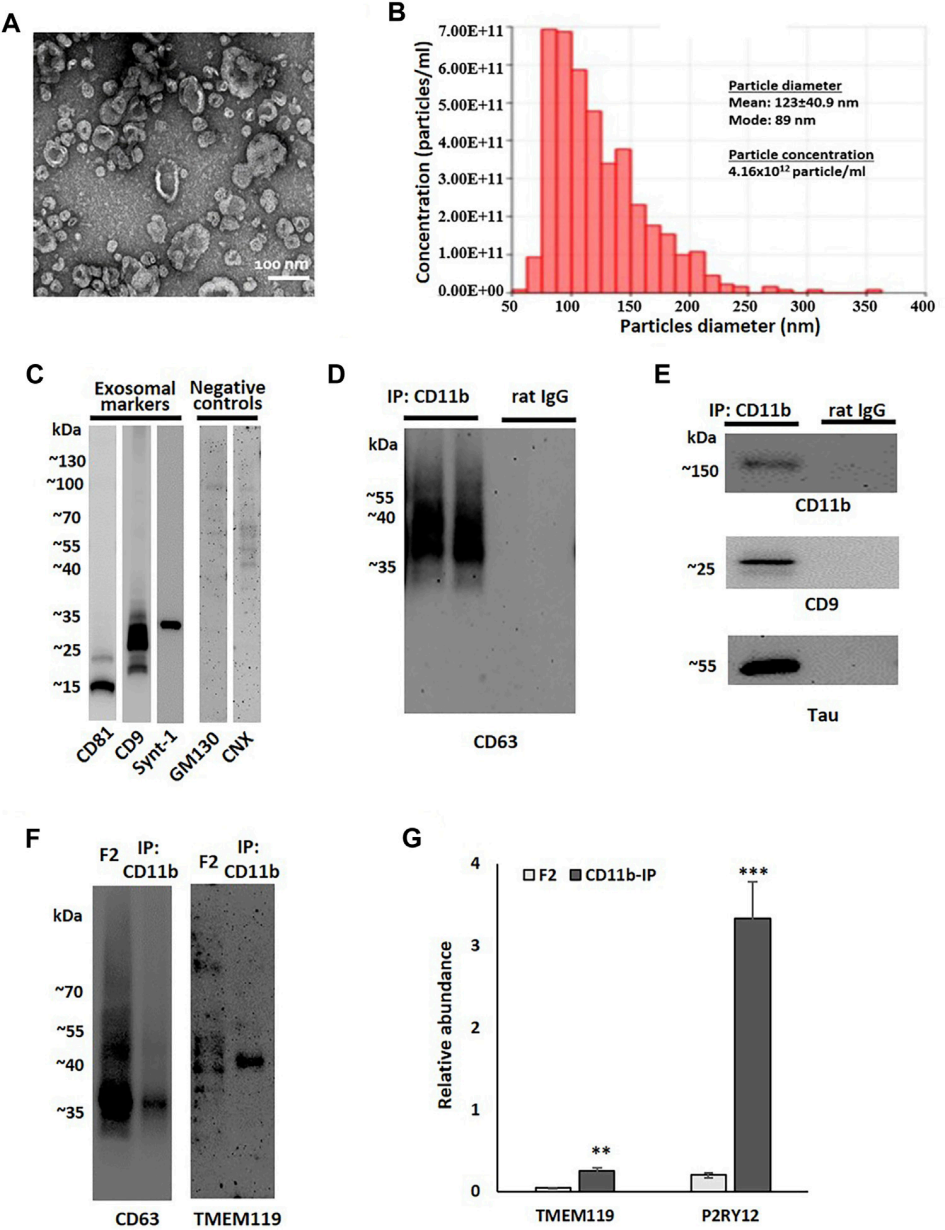
Small EV fraction characterization and microglial EV purification. **(A)** Representative transmission electron microscopy (TEM) image of small EV fraction (F2) isolated from the human parietal cortex. **(B)** Tunable resistive pulse sensing (TRPS) analysis of the F2 fraction isolated from the human parietal cortex. **(C)** A representative image of immunoblot analysis of an F2 fraction using antibodies against exosomal markers (CD9, CD81, and syntenin-1) and negative control markers (GM130 and calnexin, CNX). **(D)** A representative image of immunoblot analysis of two immunoprecipitation (IP) samples pull-down either with antibodies against microglial marker CD11b or with corresponding isotype control antibodies (rat IgG2b). Membrane was probed with the exosomal marker CD63. **(E)** Immunoblotting of CD11b-IP samples and isotype control samples probed with antibodies against a microglial marker (CD11b), an exosomal marker (CD9), and tau. **(F)** Immunoblotting of the F2 fraction isolated from the human parietal cortex and corresponded CD11b-IP fraction with antibodies against the exosomal marker CD63 and the microglial marker TMEM119. The sample volume was equal between CD63 and TMEM119 immunoblots. **(G)** Relative abundances of microglial markers, TMEM119 and P2RY12, in F2 (n = 6) and CD11b-IP samples (n = 7) as indicated by LC-MS/MS (**p < 0.001; ***p < 0.0001).

Based on a previously described method of brain EV isolation (Vella, et al., 2017), brain tissue from two late AD cases (Braak V-VI) was used for the development and standardization of the microglial EV isolation protocol. Sucrose-gradient fractions enriched in EVs were isolated from cryopreserved tissue after enzymatic digestion and ultracentrifugation. TEM and TRPS analysis confirmed the abundance of small EVs (below 200 nm in diameter) in Fraction 2 (F2) (Figures 1A,B). This fraction was enriched in exosomal markers CD9, CD81, and syntenin-1 and contained only trace amounts of negative control markers, such as Golgi complex protein GM130 and endoplasmic reticulum protein calnexin (CNX) (Thery, et al., 2018) (Figure 1C). The F2 fraction was further used for the isolation of microglial EVs by immunoprecipitation with antibodies against the microglia-specific marker CD11b. Immunoblot analysis demonstrated the enrichment of exosomal markers, CD63 and CD9, and the microglial marker, CD11B, in small EVs immunoprecipitated with CD11b-conjugated Dynabeads when compared to isotype control antibody-conjugated beads (Figures 1D,E). We also compared the levels of the microglial marker TMEM119 between the F2 fraction and the corresponding CD11b-positive microglial fraction (Figure 1F). The data demonstrate the enrichment of TMEM119 in the IP fraction despite much higher EV load in the F2 lane as documented by the CD63 signal (Figure 1F). We tested relative abundances of TMEM119 and another microglial marker P2RY12 in F2 and CD11b-IP samples by proteomics. Proteomic analysis revealed the enrichment of microglia-associated proteins in CD11b-pos EVs when compared to F2 fractions from NL and AD brain tissue. Relative to other proteins in each sample, two microglia markers, TMEM119 and P2RY12, were significantly more abundant in CD11b-purified samples with 6.1- and 16.9-fold increase, respectively (Figure 1G). Tau was detected in some but not all microglial EV samples by immunoblotting (Figure 1E); however, Aβ peptide was not detected (data not shown) either because of low levels or difficulties relating to Aβ detection without prior delipidation (Perez-Gonzalez, et al., 2017).

For the omics analyses, microglial EVs enriched from 1 g of cryopreserved human parietal cortex tissue from three normal/low pathology cases (NL 1-3) and four late AD cases (AD 1–4) were used. Demographic information for all the cases is presented in Table 2.

### Proteomic Analysis of Microglial EVs

**FIGURE 2:**
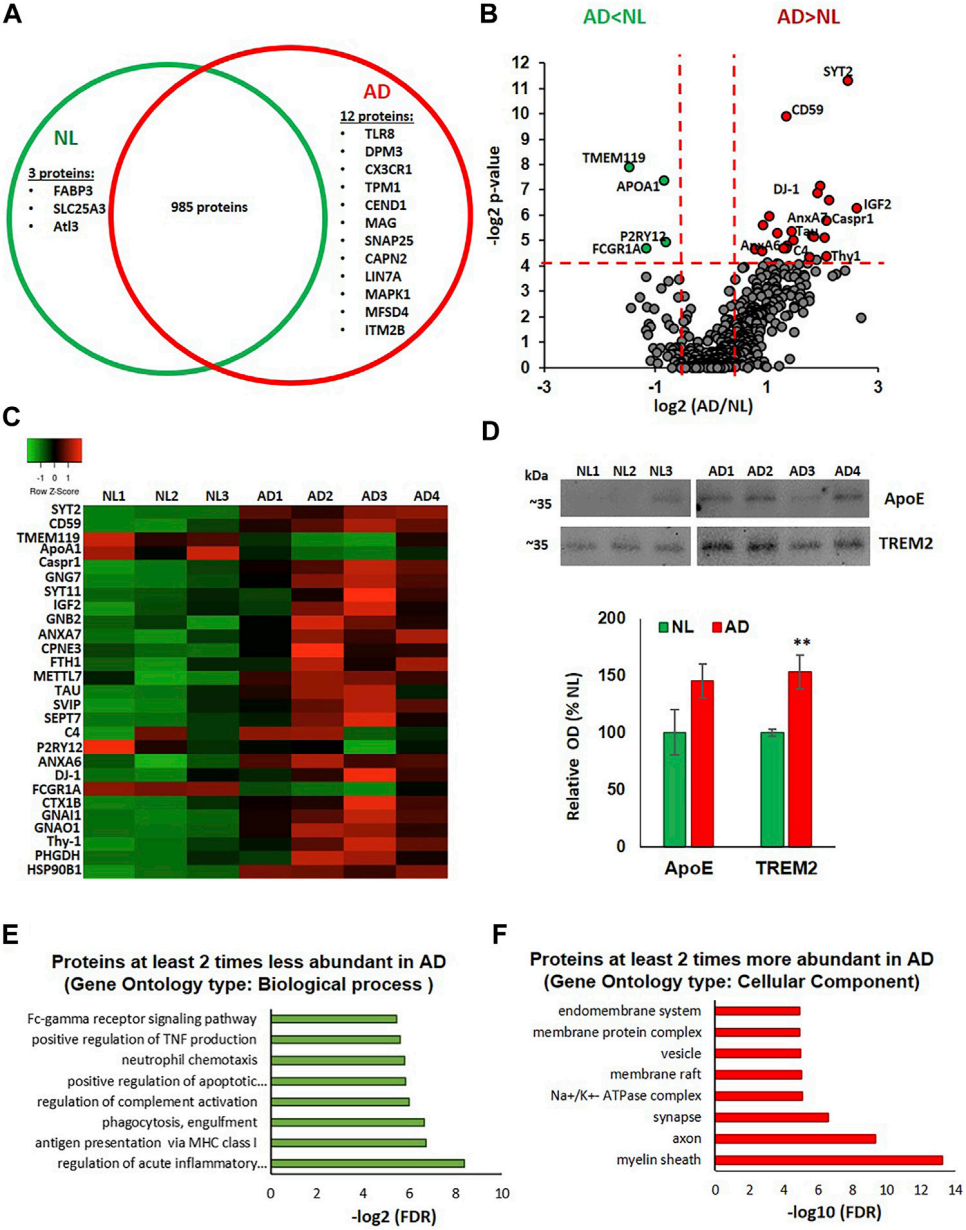
Proteomic analysis of microglial EVs from normal/low pathology and late-stage AD cases. **(A)** Venn diagram representing microglial proteins differentially detected in NL and AD groups. **(B)** Volcano plot showing a degree of differential expression of proteins in NL and AD groups. Vertical red dotted lines separate proteins, which are two or more times less abundant in AD (far left segment) and two or more times more abundant in AD (far right segment). Horizontal red dotted line separates the top part of the plot containing dots representing proteins whose abundances are significantly different between NL and AD groups (*p* < 0.05) and the bottom part (no significant changes). Proteins that are significantly different between AD and NL groups are color-coded: two or more times more abundant in AD are presented in red, two or more times less abundant in green. **(C)** Heat map of proteins significantly up- and downregulated in AD compared to the NL group across seven evaluated human cases. **(D)** Immunoblotting of CD11b-IP samples with antibodies against ApoE and TREM2. Representative images are in the upper section and the composite data are in the lower section (*p < 0.05). **(E)** Gene ontology enrichment bioinformatic analysis of proteins two or more times less abundant in AD compared to NL. **(F)** Gene ontology enrichment bioinformatic analysis of proteins two or more times more abundant in AD compared to NL.

Using Tandem Mass Tag (TMT)-based quantitative proteomics, a total of 1,000 unique proteins were identified in the microglia-derived small EV samples from the human parietal cortex. Among these, 985 were detected in both NL and AD groups. Three proteins, fatty acid binding protein 3, heart type (FABP3), mitochondrial copper transporter Solute Carrier Family 25 Member 3 (SLC25A3), and GTPase Atlastin-3(Alt3), were only detected in the NL samples. Twelve proteins were detected only in the AD samples. These included innate immune response proteins toll-like receptor 8 (TLR8) and CX3C chemokine receptor 1 (CX3CR1), neuron-specific proteins, cell cycle exit and neuronal differentiation 1 (CEND1), synaptosomal-associated protein 25 (SNAP25) and myelin-associated glycoprotein (MAG), and two known regulators of amyloid precursor protein (APP) metabolism, calpain-2 catalytic subunit (CAPN2) (Mahaman, et al., 2019) and integral membrane protein 2B (ITM2B/BRI2) (Matsuda et al., 2008) (Figure 2A). A total of 469 proteins were quantified in all analyzed samples, providing relative abundances for each protein. Differences in protein abundances are illustrated via a volcano plot, which shows that 4 proteins were significantly downregulated and 23 proteins were significantly upregulated in the AD group when compared to the NL group (Figure 2B). A heatmap showing differential expression of the significantly altered proteins across individual samples is presented in Figure 2C. The protein composition of microglia EVs from AD cases reflects the loss of the homeostatic signature of their cells of origin, with a significant decrease in the abundance of two main markers of homeostatic microglia, TMEM119 and P2RY12 (2.8-fold and 1.8-fold, respectively). Abundances of the phagocytic microglial marker FCGR1A (CD64) and a known TREM2 ligand, ApoA1, were also significantly lower in the AD group (Figures 2B,C). There was a significant upregulation of the Stage 1 DAM (proinflammatory phenotype) marker, ferritin heavy chain-1 (FTH1; 2.7-fold) (Figures 2B,C). Most proteins with significantly higher abundances in AD cases can be divided into four groups: 1) neuronal and synapse-enriched proteins: synaptotagmin-2 (SYT-2; 5.5-fold), synaptotagmin-11 (SYT11; 4.4-fold), Syntaxin-1B (STX1B; 2.5-fold), Thy1 membrane glycoprotein (Thy1; 4.2 -fold), and tau protein (3.5-fold); 2) complement regulators: complement component 4B (C4B; 2.8-fold) and CD59 (2.6-fold); 3) GTPases: septin-7 (SEPT7; 4.1-fold) and G-protein complex subunits, guanine nucleotide-binding protein G(I)/G(S)/G(O) subunit gamma-7 (GNG7; 3.8-fold), guanine nucleotide-binding protein G(I)/G(S)/G(T) subunit beta-2 (GNB2; 2-fold), guanine nucleotide-binding protein G(i) subunit alpha-1 (GNAI1; 1.7-fold), and guanine nucleotide-binding protein G(o) subunit alpha (GNAO1; 1.9-fold); and 4) members of the annexin family involved in vesicle traffic, aggregation, and membrane fusion: annexins A6 (ANXA6; 2.6-fold) and A7 (ANXA7; 4.2-fold) (Lizarbe et al., 2013). We also identified AD-related increases in toll-like receptor chaperone, heat shock protein 90B1 (HSP90B1; 3.4-fold) (Binder, 2014); antioxidant protein, DJ-1, which is coded by a gene causative for autosomal recessive Parkinson’s disease (PARK7; 2.6-fold) (Hijioka et al., 2017) and has an emerging role in regulation of immune responses (Zhang, et al., 2020); neurotrophic insulin-like growth factor 2 (IGF2; 6.2-fold); contactin associated protein 1 (Caspr-1; 3.9-fold), which reduces Aβ production (Fan, et al., 2013) and promotes release of prosurvival secreted amyloid precursor protein (APP) domain sAPPα (Tang, et al., 2020); calcium-dependent phospholipid-binding protein copine-3 (CPNE3; 1.9-fold); and D-3-phosphoglycerate dehydrogenase (PHGDH; 3.4-fold), an enzyme essential for the synthesis of l-serine (Figures 2B,C). Interestingly, brain levels of the PHGDH are decreased in AD brain samples, and extracellular PHGDH mRNA was recently proposed to be an early presymptomatic blood marker for Alzheimer’s disease (Yan, et al., 2020). Several proteins with mostly unknown functions, including small VCP/p97-interacting protein (SVIP; 3.6-fold) and methyltransferase-like protein 7A (METTL7A; 2.3-fold), were also significantly more abundant in microglial EVs from the AD brain (Figures 2B,C).

Notably, the abundance of ApoE, a Stage 1 DAM marker, was 1.4 times higher in microglial EVs from the AD brain when compared to NL, although this difference was not statistically significant. We subsequently confirmed this result using immunoblotting (Figure 2D), which also revealed an ∼40% increase in ApoE levels in the AD group, validating the changes observed in the proteomics analysis. To our surprise, the mass spectrometric analysis did not detect the microglia marker TREM2. In contrast, TREM2 was detected in the same samples using immunoblotting, and average TREM2 levels were significantly higher in the AD group (Figure 2D **).** Interestingly, amyloid-beta precursor protein (APP) was detected in all samples with only one unique peptide (AA 578-589) that corresponds to a region of Aβ. The abundance of the APP/Aβ was 1.9 times higher in the AD group when compared to NL, but this difference was not statistically significant (data not shown).

The proteomic data set was further analyzed using the STRING database for enrichment gene ontology (GO) and pathway analysis. Proteins exhibiting a decrease of twofold or greater in the AD group were enriched in several immune regulation pathways, including acute inflammatory response, antigen presentation, phagocytosis, complement regulation, TNF production, and Fc-gamma receptor signaling (Figure 2E). Proteins exhibiting an increase of twofold or greater in the AD group showed enrichment in the following cellular components: myelin sheath, synaptic, and endosomal vesicular related proteins (Figure 2F).

### miRNA Profiling of Microglial EVs

**FIGURE 3:**
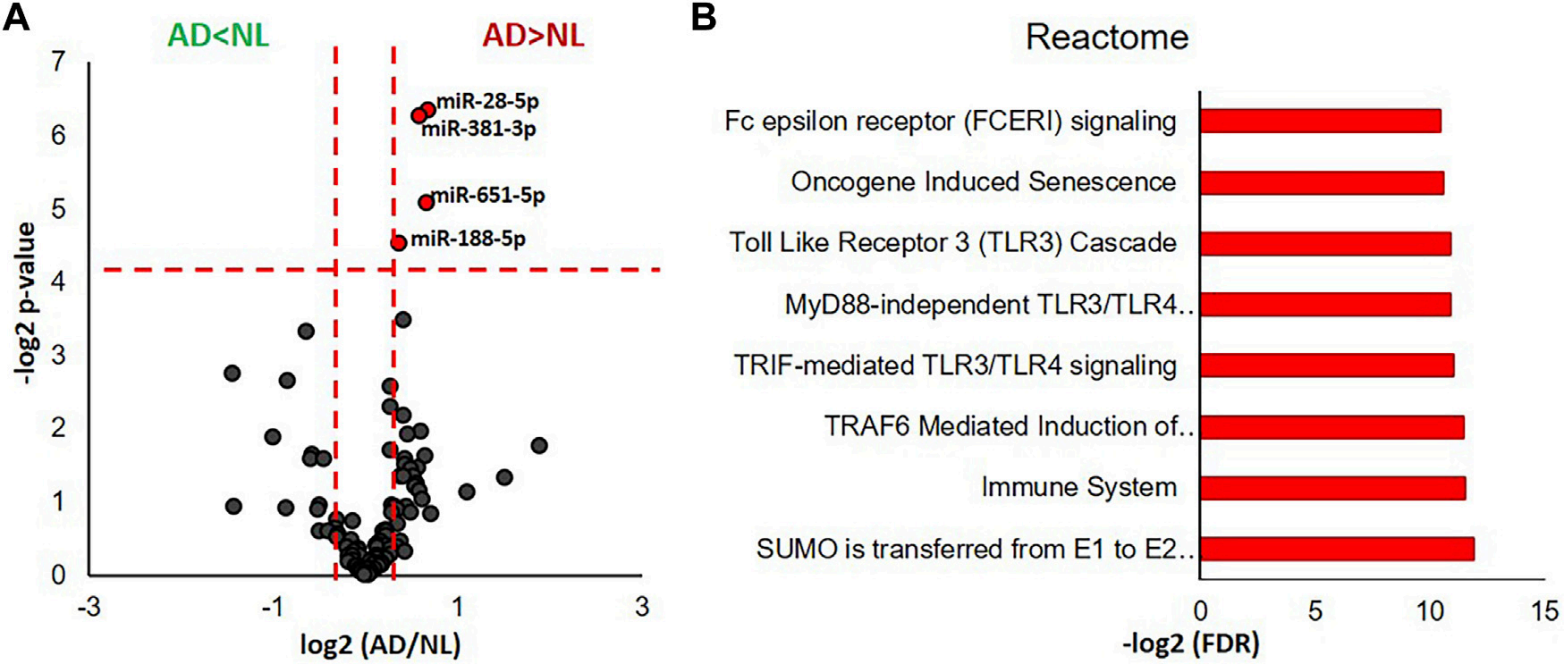
miRNA transcriptomic analysis of microglial EVs from normal/low pathology and late-stage AD cases. **(A)** Volcano plot showing a degree of differential expression of miRNAs in NL and AD groups. Vertical red dotted lines separate miRNA, which are 1.5 or more times less abundant in AD (far left segment) and 1.5 or more times more abundant in AD (far right segment). Horizontal red dotted line separates the top section of the plot containing dots representing proteins whose abundance is significantly different between NL and AD groups (*p < 0.05). MicroRNAs, which are significantly different between AD and NL groups, are color-coded: 1.5 or more times more abundant in AD are presented in red, 1.5 or more times less abundant are in green. **(B)** Gene ontology enrichment bioinformatic analysis of miRNAs significantly more abundant in AD compared to NL.

We identified 105 miRNAs present in 5 or more of the analyzed human cases using the nCounter miRNA expression panel. Fold changes (AD/NL) and *p*-values corresponding to each identified miRNA were illustrated by a volcano plot, which revealed that levels of four miRNA—miR-28-5p, miR-381-3p, miR-651-5p, and miR-188-5p—were significantly higher in microglial EVs from AD cases when compared to NL cases (Figure 3A). Functional interpretation of these data was performed using miRNet (Fan, et al., 2016), which revealed SUMOylation, toll-like receptor (TLR), Fc epsilon receptor I (FCERI), and senescence pathways to be among those regulated by the four significantly increased miRNAs (Figure 3B).

### Lipidomics of Microglial EVs

**FIGURE 4:**
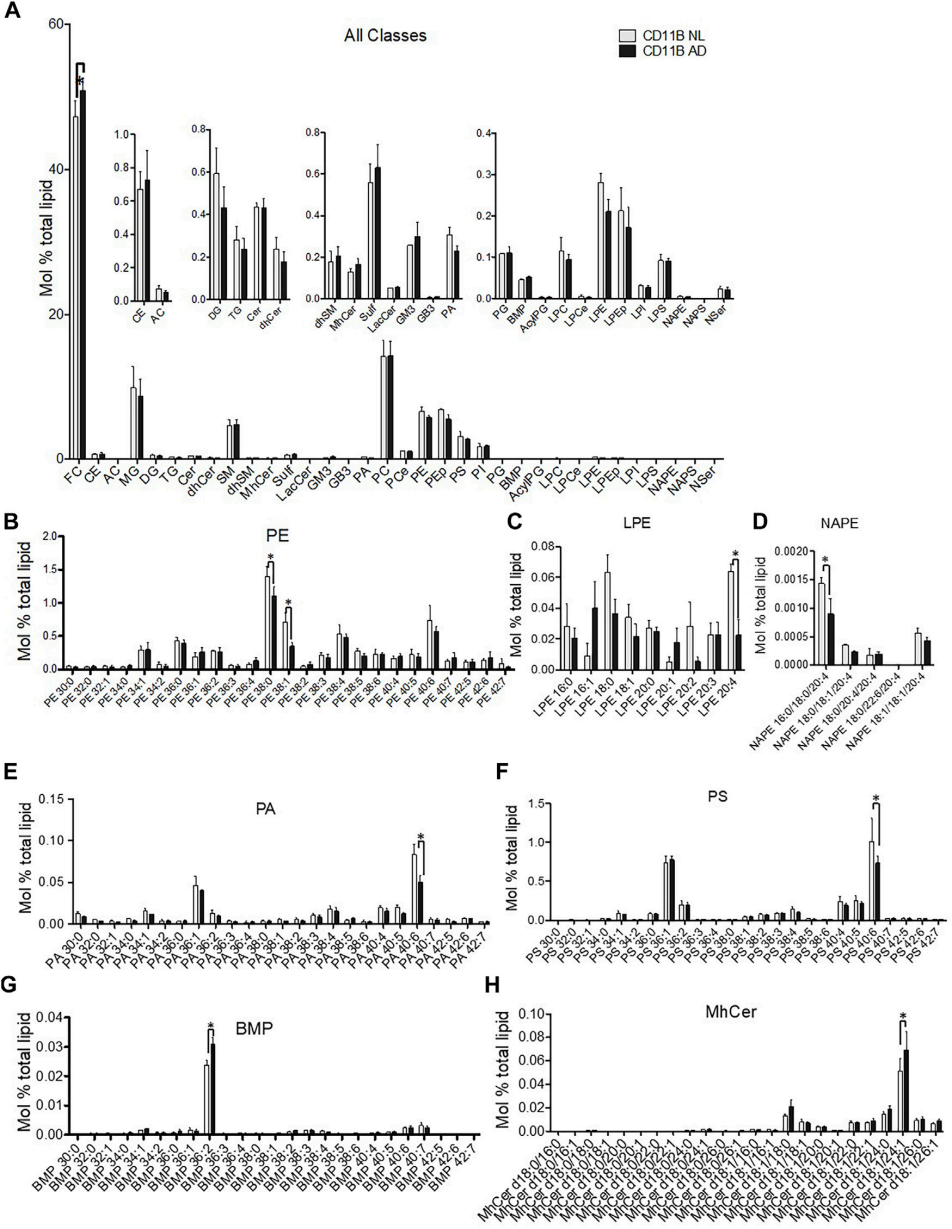
Lipidomic analysis of microglial EVs from normal/low pathology and late-stage AD cases. **(A)** Relative quantification 34 lipid classes and 593 individual lipid species in microglial EVs. Lipids are quantified as mol% total lipid by normalizing the molar amount of each lipid species to the summed total moles of all lipid species in each sample. The inset panels show lower abundance species on the expanded *y*-axis. Abbreviations are free cholesterol (FC), cholesteryl ester (CE), acyl carnitine (AC), monoacylglycerol (MG), diacylglycerol (DG), triacylglycerol (TG), ceramide (Cer), sphingomyelin (SM), monohexosylceramide (MhCer), sulfatides (Sulf), lactosylceramide (LacCer), monosialodihexosylganglioside (GM3), globotriaosylceramide (GB3), phosphatidic acid (PA), phosphatidylcholine (PC), ether phosphatidylcholine (PCe), phosphatidylethanolamine (PE), plasmalogen phosphatidylethanolamine (Pep), phosphatidylserine (PS), phosphatidylinositol (PI), phosphatidylglycerol (PG), bis(monoacylglycerol)phosphate (BMP), acyl phosphatidylglycerol (AcylPG), lysophosphatidylcholine (LPC), ether lysophosphatidylcholine (LPCe), lysophosphatidylethanolamine (LPE), plasmalogen lysophosphatidylethanolamine (LPEp), lysophosphatidylinositol (LPI), lysophosphatidylserine (LPS), N-Acyl phosphatidylethanolamine (NAPE), N-acyl phosphatidylserine (NAPS), and N-acyl serine (NSer). Data are presented as mean mol% with error bars showing mean ± S.E. FC is significantly increased shown in the primary panel (*p* < 0.01). **(B)** PE species were detected using mass spectrometry and are shown using relative quantification as mol%. PE 38:0 (*p* < 0.05); PE 38:1 (*p* < 0.01). **(C)** Most abundant LPE [LPE20:4 (*p* < 0.05)] and **(D)** NAPE [NAPE 16:0/18:0/20:4 (*p* < 0.05)] lipid species are shown to significantly differ between LN- and AD-derived microglia EV. **(E)** PA40:6 lipid species is decreased in microglia-derived EV from the AD brain (AD) compared to control (LN) (*p* < 0.0001), while no other PA species are significantly affected. **(F)** The most abundant PS species, PS40:6 species, is decreased in EV derived from the AD brain (AD) compared to control (LN) (*p* < 0.001) while no other PS species are significantly altered. **(G)** The most abundant BMP 36:2 (*p* < 0.0001) and **(H)** MhCer d18:1/24:1 (*p* < 0.01) lipid species are increased in AD-derived microglia EV. All data are presented as mean mol% with error bars showing mean ± S.E.

Analysis across all lipid classes without regard to acyl chain composition indicated that only free cholesterol (FC) was increased in microglia-derived EVs from the AD brain (Figure 4A). Phospholipids showed acyl chain specific changes in mono- and polyunsaturated species. Microglia-derived EVs from the AD brain showed a significant deficit in phosphatidylethanolamine (PE) 38:0 and 38:1 (Figure 4B) and a concurrent loss of the PE metabolites—lysoPE 2:4 and N-acyl phosphatidylethanolamine (NAPE) 16:0/18:0/20:4 (Figures 4C,D). Interestingly, differences in microglia-derived EVs from the AD brain were seen on phospholipids likely to harbor docosahexaenoic acid (22:6) in the sn-2 position. Specifically, microglia-derived EVs showed significant deficits in phosphatidic acid (PA) 40:6 (Figure 4E) and phosphatidylserine (PS) 40:6 (Figure 4F). A trend toward depletion of 40:6 was also detected in PE (PE 40:6) (Figure 4B) and PEp (PEp40:6) (data not shown). Specific lipid changes observed in microglial EVs included upregulation of the most abundant lipid species of BMP and monohexosylceramides (mhCer). The most abundant BMP species BMP 36:2 was enriched in EVs derived from the AD brain (Figure 4G). Finally, the most abundant mhCer, mhCer d18:1/24:1, was significantly upregulated in microglial-derived EVs from the AD brain (Figure 4H).

## Discussion

We evaluated the changes across the proteome, lipidome, and miRNA transcriptome of small EVs of microglial origin (positive for myeloid cell marker, CD11B) isolated from the human parietal cortex of three normal/low pathology (NL) and four late-stage AD cases (Table 2).

We found a significant decrease in the abundance of known homeostatic microglial markers, P2RY12 and TMEM119, and a corresponding increase in DAM markers, FTH1 (Stage 1 DAM) and TREM2 (Stages 1 and 2 DAM), in microglial EVs from the AD brain when compared to NL. However, a homeostatic microglia marker, Cx3CR1, was detected in AD, but not in the NL group, and a classical Stage 1 DAM marker, ApoE, was only marginally increased in AD (Figures 2A–D,). Overall, these changes better recapitulate the overall brain cell transcriptional signatures of DAM from mouse AD models (Deczkowska, et al., 2018), which were not reproduced in a recent human RNAseq study (Srinivasan, et al., 2020). A possible explanation for the discrepancy can be disproportional enrichment of DAM-derived EVs in the microglial secretome from the AD tissue. The burdens of cortical dense-core plaques in AD mouse models (Tg2576, APP_Swe_PS1ΔE9, and 5XFAD) greatly exceed the cortical plaque burdens in human AD cases (P. Liu, et al., 2017). As a result, the proportion of DAM cells surrounding dense-core plaques (Keren-Shaul, et al., 2017) may be higher in the mouse models when compared with the human disease, and a DAM-specific transcriptomic signature may be easier to detect. It has been recently demonstrated that small EV secretion is highly upregulated in mouse DAM cells (Clayton, et al., 2021). Moreover, mouse and human DAM display a transcriptional signature characteristic of senescent cells (Hu, et al., 2021), which are known to secrete vast numbers of EVs as a part of the senescence-associated secretory phenotype (SASP). In agreement with this, our miRNA transcriptome data revealed senescence as one of the top pathways controlled by four miRNAs identified to be significantly upregulated in AD microglial EVs (Figure 3B). Thus, it is possible that analysis of human microglial EVs may more specifically address changes in the Aβ-plaque-associated DAM population when compared to cell RNAseq analysis.

Microglial EVs either contain low levels of tau protein or a composition of tau species that cannot be easily detected by immunoblotting analysis. Yet, tau was detected in all samples by mass spectrometry, and tau abundance was significantly higher in AD when compared to NL samples (Figures 2B,C). These data confirmed a potential role of microglia-derived EVs in the spread of tau pathology in the human AD brain, as it was previously described *in vitro* and in mouse tauopathy and AD models (Asai, et al., 2015; Clayton, et al., 2021; Maphis, et al., 2015). The specific microglial EV-associated tau species and their proteopathic seeding potential still need to be defined.

In addition to the increase in abundance of tau protein in AD brain-derived microglial EVs, we found that levels of neuron-specific and synapse-enriched proteins were either exclusively detected (CEND1 and SNAP25) or significantly upregulated (SYT-1, SYT-11, STX1B, and Thy1) in the AD group (Figures 2A,B). Myelin-specific protein MAG was only found in microglial EVs from AD, but not in NL samples (Figure 2A). Moreover, myelin sheath was the cellular component most enriched in proteins that were increased twofold or greater in the AD group (Figure 2F). Increases in synaptic protein VGlut1 in microglial cells from the 5xFAD mouse model and in TDP43-depleated microglia with a hyperphagocytic phenotype have been described (Brioschi, et al., 2020), so it is likely that synaptic and myelin-specific proteins have been phagocytosed prior to entering microglial exosomes. In this regard, elevated levels of complement protein, C4, in microglial EVs from AD cases (Figure 2B) may suggest that the process of complement-dependent engulfment of synapses by microglia may be involved (Thion and Garel, 2018; Yilmaz, et al., 2021). On the other hand, C4-coated EVs may play a role as scavengers and thus may protect cells from complement attack (Karasu et al., 2018). Interestingly, we also observed an increase in membrane attack complex (MAC)-inhibitory protein, CD59, which is known to negatively regulate complement-mediated phagocytosis (Schartz and Tenner, 2020; Tenner, 2020).

Our lipidomic analysis revealed an increase in cholesterol in AD microglial EVs (Figure 4A), which is consistent with the phagocytosis of neuronal debris by microglial cells. Cholesterol metabolism has also been associated with immune activation (Deczkowska, et al., 2018; Orre, et al., 2014; Simon, 2014; Spann and Glass, 2013; Wong, et al., 2020). A recent study reported cholesteryl ester accumulation in TREM2-deficient microglia that fails to mount an immune response (Nugent, et al., 2020). However, we did not find significant changes in CE species levels in microglia-derived EVs in the AD cases reported here.

We also found a significant increase in the major bis(monoacylglycerol)phosphate (BMP) and monohexosylceramide (MhCer) lipid species (Figures 4G,H). An increase of this species of BMP, a lysosome-specific lipid, suggests increased lysosomal lipid content in AD microglia (Showalter, et al., 2020). Elevated levels of MhCer may play a role in microglial activation and immune response (Brennan, et al., 2017; Miltenberger-Miltenyi, et al., 2020; Niimura et al., 2010), as well as be a precursor of more complex gangliosides (Merrill, 2011). Interestingly, enrichment of BMP and MhCer in EVs has been previously linked to the impairment of endolysosomal function induced by Vsp34 kinase inhibition, which causes phosphatidylinositol-3-phosphate (PI3P) deficiency (Miranda, et al., 2018). Thus, endolysosomal impairment can explain the elevated levels of neuronal and myelin molecules in microglial EVs from the AD brain and potentially lead to the disruption of microglial pathways as suggested by the bioinformatic analysis in Figure 2E.

We observed that a reduction in phospholipids is likely to harbor docosahexaenoic acid (DHA, 22:6) in the sn-2 position in microglial EVs from the AD brain. One likely acyl chain configuration for PS40:6 and PA40:6 is sn-1 18:0, sn-2 22:6. These data echo the previously described DHA decrease in the bulk preparations of human AD brain EVs and global AD-related brain DHA deficiency, which has a proinflammatory effect and may also disrupt Aβ clearance (H. Su, et al., 2021). The selective loss of DHA among PA and PS (Figures 4E,F) as well as the trend in PE (Figure 2B) suggests the overactivation of DHA-selective phospholipase A 2 (PLA2) and a deficit in acyl chain remodeling (Abdullah, et al., 2017; Calon, et al., 2004; Fernandez, et al., 2018; Fernandez, et al., 2021; Fonteh, et al., 2013; Granger, et al., 2019).

The loss of LPE20:4 in EVs derived from AD microglia (Figure 4C) may represent exhaustion of acyl chain remodeling of PE. Since 20:4 is cleaved from PE20:4 to liberate the free fatty acid, arachidonate, it may represent loss of this proinflammatory fatty acid due to overactivation of PLA2, which has previously been reported in AD (Sanchez-Mejia, et al., 2008; Wang, et al., 2021). Interestingly, NAPE is an important precursor to endocannabinoid synthesis, which is dysregulated in AD (Bisogno and Di Marzo, 2008; Fonteh, et al., 2013; Liu, et al., 2006). Even though previous studies have reported global loss of PE and plasmalogen, our analyses show only a trend for PE depletion and no loss in plasmalogen from AD brain microglia-derived EVs (Han et al., 2001; Han et al., 2002).

We did not find AD-associated changes in key modulators of immune responses—miR-146a-5p, miR-155-5p, and miR-124-3p (Su et al., 2016); miR-124-3p was not detected in our samples, while miR-146a-5p and miR-155-5p levels were above background but did not show any significant differences between NL and AD groups (data not shown). Two out of four miRNAs upregulated in the AD group, miR-188-5p and miR-381-3p (Figure 3A), are known to have neuroprotective effects. MiR-188-5p restores synaptic and cognitive deficits in 5xFAD mice (Lee, et al., 2016), and miR-381-3p promotes the recovery of spinal cord injury in rats (Chen et al., 2018). In addition to a neuroprotective miRNA signature, proteomics revealed increases in the abundance of IGF2, ITM2B and CASPR-1, and DJ-1 in microglial EVs from AD cases (Figures 2A–B). Neurotrophic factor IGF2 was shown to reduce Aβ amyloidosis, reverse synaptic deficits, and improve memory in AD animal models (Mellott et al., 2014; Pascual-Lucas, et al., 2014). ITM2B and CASPR-1 are known to regulate APP metabolism and suppress Aβ production (Fan, et al., 2013; Matsuda, et al., 2008; Tang, et al., 2020), and DJ-1 is an important antioxidant with newly discovered immune regulation functions (Zhang, et al., 2020). The presence of both neuroprotective and pathology-related miRNA and proteins in AD cases, a seemingly contradictory result, may represent both a consequence of, and a response to, disease pathogenesis. It is interesting to surmise that the response and consequence may be present as the cargo in different EV subpopulations, either derived from the same cells or from microglia at different stages of activation. Further evaluation of EV subpopulations and the molecular mechanisms dictating their release may provide a new avenue for AD therapeutic development with the aim to suppress subpopulations containing pathogenic cargo, while promoting the release of neuroprotective EVs.

In conclusion, our data suggest that loss of a homeostatic signature and the deterioration of functional microglia in late AD stages may accompany endolysosomal impairment and the release of undigested neuronal and myelin debris, including tau, through extracellular vesicles. We also found a significant AD-associated decrease in levels of DHA-containing polyunsaturated lipids of different classes, which may be associated with global DHA deficiency in AD and indicate a potential defect in the acyl-chain remodeling by PLA2 and lysophospholipid acyltransferases. On the other hand, the AD-specific microglial EV signature also includes increases in some miRNAs and proteins with neuroprotective properties. It is currently unknown if those “harmful” vs. “protective” molecular signatures represent different subpopulations of microglial EVs. Results from our study support the hypothesis that the molecular composition of EVs reflects functional changes in microglia consistent with a diseased state. To the best of our knowledge, this is the first study analyzing proteins, lipids, and miRNAs in cell-type specific EVs from human brain tissue. The main limitation of the study is the small number of cases, and our results require further validation in larger cohorts, and when possible inclusion of samples from earlier disease stages. Despite these limitations, this proof-of-concept study clearly demonstrates the feasibility of using multiple omics analyses on small microglial EVs isolated from cryopreserved human brain tissue. Our results exemplify the superiority of an integrative approach when compared to individual proteomics, lipidomics, or microRNA analyses, and suggest that new AD biomarkers may arise from all three different classes of biomolecules.

## Data Availability

The datasets presented in this study can be found in online repositories. The names of the repository/repositories and accession number(s) can be found below: PRIDE, accession no: PXD028898.

## References

[B1] 2021 Alzheimer's disease facts and figures, 2021 2021 Alzheimer's disease facts and figures (2021). Alzheimers Dement 17, 327–406. 10.1002/alz.12328 33756057

[B2] Abdullah L. Evans J. E. Emmerich T. Crynen G. Shackleton B. Keegan A. P. (2017). APOE ε4 Specific Imbalance of Arachidonic Acid and Docosahexaenoic Acid in Serum Phospholipids Identifies Individuals with Preclinical Mild Cognitive Impairment/Alzheimer's Disease. Aging (Albany NY) 9, 964–985. 10.18632/aging.101203 28333036 PMC5391242

[B3] An K. Klyubin I. Kim Y. Jung J. H. Mably A. J. O'Dowd S. T. (2013). Exosomes Neutralize Synaptic-Plasticity-Disrupting Activity of Aβ Assemblies *In Vivo* . Mol. Brain 6, 47. 10.1186/1756-6606-6-47 24284042 PMC4222117

[B4] Asai H. Ikezu S. Tsunoda S. Medalla M. Luebke J. Haydar T. (2015). Depletion of Microglia and Inhibition of Exosome Synthesis Halt Tau Propagation. Nat. Neurosci. 18, 1584–1593. 10.1038/nn.4132 26436904 PMC4694577

[B5] Bilousova T. Elias C. Miyoshi E. Alam M. P. Zhu C. Campagna J. (2018). Suppression of Tau Propagation Using an Inhibitor that Targets the DK-Switch of nSMase2. Biochem. Biophys. Res. Commun. 499, 751–757. 10.1016/j.bbrc.2018.03.209 29604274 PMC5956110

[B6] Bilousova T. Simmons B. J. Knapp R. R. Elias C. J. Campagna J. Melnik M. (2020). Dual Neutral Sphingomyelinase-2/Acetylcholinesterase Inhibitors for the Treatment of Alzheimer's Disease. ACS Chem. Biol. 15, 1671. 10.1021/acschembio.0c00311 32352753 PMC8297715

[B7] Binder R. J. (2014). Functions of Heat Shock Proteins in Pathways of the Innate and Adaptive Immune System. J. Immunol. 193, 5765–5771. 10.4049/jimmunol.1401417 25480955 PMC4304677

[B8] Bisogno T. Di Marzo V. (2008). The Role of the Endocannabinoid System in Alzheimer's Disease: Facts and Hypotheses. Curr. Pharm. Des. 14, 2299–3305. 10.2174/138161208785740027 18781980

[B9] Bligh E. G. Dyer W. J. (1959). A Rapid Method of Total Lipid Extraction and Purification. Can. J. Biochem. Physiol. 37, 911–917. 10.1139/o59-099 13671378

[B10] Bosch S. de Beaurepaire L. Allard M. Mosser M. Heichette C. Chrétien D. (2016). Trehalose Prevents Aggregation of Exosomes and Cryodamage. Sci. Rep. 6, 36162. 10.1038/srep36162 27824088 PMC5099918

[B11] Brennan P. J. Cheng T. Y. Pellicci D. G. Watts G. F. M. Veerapen N. Young D. C. (2017). Structural Determination of Lipid Antigens Captured at the CD1d-T-Cell Receptor Interface. Proc. Natl. Acad. Sci. U S A. 114, 8348–8353. 10.1073/pnas.1705882114 28716901 PMC5547638

[B12] Brioschi S. d'Errico P. Amann L. S. Janova H. Wojcik S. M. Meyer-Luehmann M. (2020). Detection of Synaptic Proteins in Microglia by Flow Cytometry. Front. Mol. Neurosci. 13, 149. 10.3389/fnmol.2020.00149 33132837 PMC7550663

[B13] Brumbaugh C. D. Kim H. J. Giovacchini M. Pourmand N. (2011). NanoStriDE: Normalization and Differential Expression Analysis of NanoString nCounter Data. BMC Bioinformatics. 12, 479. 10.1186/1471-2105-12-479 22177214 PMC3273488

[B14] Butovsky O. Weiner H. L. (2018). Microglial Signatures and Their Role in Health and Disease. Nat. Rev. Neurosci. 19, 622–635. 10.1038/s41583-018-0057-5 30206328 PMC7255106

[B15] Calon F. Lim G. P. Yang F. Morihara T. Teter B. Ubeda O. (2004). Docosahexaenoic Acid Protects from Dendritic Pathology in an Alzheimer's Disease Mouse Model. Neuron 43, 633–645. 10.1016/j.neuron.2004.08.013 15339646 PMC2442162

[B16] Chan R. B. Oliveira T. G. Cortes E. P. Honig L. S. Duff K. E. Small S. A. (2012). Comparative Lipidomic Analysis of Mouse and Human Brain with Alzheimer Disease. J. Biol. Chem. 287, 2678–2688. 10.1074/jbc.M111.274142 22134919 PMC3268426

[B17] Chen W. C. Luo J. Cao X. Q. Cheng X. G. He D. W. (2018). Overexpression of miR-381-3p Promotes the Recovery of Spinal Cord Injury. Eur. Rev. Med. Pharmacol. Sci. 22, 5429–5437. 10.26355/eurrev_201809_15802 30229813

[B18] Cheng L. Vella L. J. Barnham K. J. McLean C. Masters C. L. Hill A. F. (2020). Small RNA Fingerprinting of Alzheimer's Disease Frontal Cortex Extracellular Vesicles and Their Comparison with Peripheral Extracellular Vesicles. J. Extracell Vesicles 9, 1766822. 10.1080/20013078.2020.1766822 32922692 PMC7448944

[B19] Clayton K. Delpech J. C. Herron S. Iwahara N. Ericsson M. Saito T. (2021). Plaque Associated Microglia Hyper-Secrete Extracellular Vesicles and Accelerate Tau Propagation in a Humanized APP Mouse Model. Mol. Neurodegener 16, 18. 10.1186/s13024-021-00440-9 33752701 PMC7986521

[B20] Crotti A. Sait H. R. McAvoy K. M. Estrada K. Ergun A. Szak S. (2019). BIN1 Favors the Spreading of Tau via Extracellular Vesicles. Sci. Rep. 9, 9477. 10.1038/s41598-019-45676-0 31263146 PMC6603165

[B21] D'Anca M. Fenoglio C. Serpente M. Arosio B. Cesari M. Scarpini E. A. (2019). Exosome Determinants of Physiological Aging and Age-Related Neurodegenerative Diseases. Front. Aging Neurosci. 11, 232. 10.3389/fnagi.2019.00232 31555123 PMC6722391

[B22] Deczkowska A. Keren-Shaul H. Weiner A. Colonna M. Schwartz M. Amit I. (2018). Disease-Associated Microglia: A Universal Immune Sensor of Neurodegeneration. Cell 173, 1073–1081. 10.1016/j.cell.2018.05.003 29775591

[B23] Del-Aguila J. L. Li Z. Dube U. Mihindukulasuriya K. A. Budde J. P. Fernandez M. V. (2019). A Single-Nuclei RNA Sequencing Study of Mendelian and Sporadic AD in the Human Brain. Alzheimers Res. Ther. 11, 71. 10.1186/s13195-019-0524-x 31399126 PMC6689177

[B24] Dinkins M. B. Dasgupta S. Wang G. Zhu G. Bieberich E. (2014). Exosome Reduction *In Vivo* Is Associated with Lower Amyloid Plaque Load in the 5XFAD Mouse Model of Alzheimer's Disease. Neurobiol. Aging 35, 1792–1800. 10.1016/j.neurobiolaging.2014.02.012 24650793 PMC4035236

[B25] Dodd P. R. Hardy J. A. Baig E. B. Kidd A. M. Bird E. D. Watson W. E. (1986). Optimization of Freezing, Storage, and Thawing Conditions for the Preparation of Metabolically Active Synaptosomes from Frozen Rat and Human Brain. Neurochem. Pathol. 4, 177–198. 10.1007/BF02834357 3561893

[B26] Fan L. F. Xu D. E. Wang W. H. Yan K. Wu H. Yao X. Q. (2013). Caspr Interaction with Amyloid Precursor Protein Reduces Amyloid-β Generation *In Vitro* . Neurosci. Lett. 548, 255–260. 10.1016/j.neulet.2013.05.055 23748076

[B27] Fan Y. Siklenka K. Arora S. K. Ribeiro P. Kimmins S. Xia J. (2016). miRNet - Sissecting miRNA-Target Interactions and Functional Associations Through Network-Based Visual Analysis. Nucleic Acids Res. 44, W135–W141. 10.1093/nar/gkw288 27105848 PMC4987881

[B28] Fernandez R. F. Kim S. Q. Zhao Y. Foguth R. M. Weera M. M. Counihan J. L. (2018). Acyl-CoA Synthetase 6 Enriches the Neuroprotective omega-3 Fatty Acid DHA in the Brain. Proc. Natl. Acad. Sci. U S A. 115, 12525–12530. 10.1073/pnas.1807958115 30401738 PMC6298081

[B29] Fernandez R. F. Pereyra A. S. Diaz V. Wilson E. S. Litwa K. A. Martínez-Gardeazabal J. (2021). Acyl-CoA Synthetase 6 Is Required for Brain Docosahexaenoic Acid Retention and Neuroprotection during Aging. JCI Insight 6. 10.1172/jci.insight.144351 PMC8262339 34100386

[B30] Fiandaca M. S. Kapogiannis D. Mapstone M. Boxer A. Eitan E. Schwartz J. B. (2015). Identification of Preclinical Alzheimer's Disease by a Profile of Pathogenic Proteins in Neurally Derived Blood Exosomes: A Case-Control Study. Alzheimers Dement 11, 600–e1. 10.1016/j.jalz.2014.06.008 25130657 PMC4329112

[B31] Fonteh A. N. Chiang J. Cipolla M. Hale J. Diallo F. Chirino A. (2013). Alterations in Cerebrospinal Fluid Glycerophospholipids and Phospholipase A2 Activity in Alzheimer's Disease. J. Lipid Res. 54, 2884–2897. 10.1194/jlr.M037622 23868911 PMC3770101

[B32] Goetzl E. J. Goetzl L. Karliner J. S. Tang N. Pulliam L. (2016). Human Plasma Platelet-Derived Exosomes: Effects of Aspirin. FASEB J. 30, 2058–2063. 10.1096/fj.201500150R 26873936 PMC4836374

[B33] Goetzl E. J. Mustapic M. Kapogiannis D. Eitan E. Lobach I. V. Goetzl L. (2016). Cargo Proteins of Plasma Astrocyte-Derived Exosomes in Alzheimer's Disease. FASEB J. 30, 3853–3859. 10.1096/fj.201600756R 27511944 PMC5067254

[B34] Goetzl E. J. Schwartz J. B. Abner E. L. Jicha G. A. Kapogiannis D. (2018). High Complement Levels in Astrocyte-Derived Exosomes of Alzheimer Disease. Ann. Neurol. 83, 544–552. 10.1002/ana.25172 29406582 PMC5867263

[B35] Granger M. W. Liu H. Fowler C. F. Blanchard A. P. Taylor M. W. Sherman S. P. M. (2019). Distinct Disruptions in Land's Cycle Remodeling of Glycerophosphocholines in Murine Cortex Mark Symptomatic Onset and Progression in Two Alzheimer's Disease Mouse Models. J. Neurochem. 149, 499–517. 10.1111/jnc.14560 30040874

[B36] Guan Z. Li S. Smith D. C. Shaw W. A. Raetz C. R. (2007). Identification of N-Acylphosphatidylserine Molecules in Eukaryotic Cells. Biochemistry 46, 14500–14513. 10.1021/bi701907g 18031065 PMC2535763

[B37] Gylys K. H. Bilousova T. (2017). Flow Cytometry Analysis and Quantitative Characterization of Tau in Synaptosomes from Alzheimer's Disease Brains. Methods Mol. Biol. 1523, 273–284. 10.1007/978-1-4939-6598-4_16 27975256 PMC5755961

[B38] Han X. Holtzman D. M. McKeel D. W. Jr. (2001). Plasmalogen Deficiency in Early Alzheimer's Disease Subjects and in Animal Models: Molecular Characterization Using Electrospray Ionization Mass Spectrometry. J. Neurochem. 77, 1168–1180. 10.1046/j.1471-4159.2001.00332.x 11359882

[B39] Han X. M Holtzman D. McKeel D. W. Jr. Kelley J. Morris J. C. (2002). Substantial Sulfatide Deficiency and Ceramide Elevation in Very Early Alzheimer's Disease: Potential Role in Disease Pathogenesis. J. Neurochem. 82, 809–818. 10.1046/j.1471-4159.2002.00997.x 12358786

[B40] Heinzelman P. Bilousova T. Campagna J. John V. (2016). Nanoscale Extracellular Vesicle Analysis in Alzheimer's Disease Diagnosis and Therapy. Int. J. Alzheimers Dis. 2016, 8053139. 10.1155/2016/8053139 27213078 PMC4861781

[B41] Hijioka M. Inden M. Yanagisawa D. Kitamura Y. (2017). DJ-1/PARK7: A New Therapeutic Target for Neurodegenerative Disorders. Biol. Pharm. Bull. 40, 548–552. 10.1248/bpb.b16-01006 28458339

[B42] Hornung S. Dutta S. Bitan G. (2020). CNS-derived Blood Exosomes as a Promising Source of Biomarkers: Opportunities and Challenges. Front. Mol. Neurosci. 13, 38. 10.3389/fnmol.2020.00038 32265650 PMC7096580

[B43] Hsu F. F. Turk J. Shi Y. Groisman E. A. (2004). Characterization of Acylphosphatidylglycerols from *Salmonella typhimurium* by Tandem Mass Spectrometry with Electrospray Ionization. J. Am. Soc. Mass. Spectrom. 15, 1–11. 10.1016/j.jasms.2003.08.006 14698549

[B44] Hu Y. Fryatt G. L. Ghorbani M. Obst J. Menassa D. A. Martin-Estebane M. (2021). Replicative Senescence Dictates the Emergence of Disease-Associated Microglia and Contributes to Aβ Pathology. Cell Rep 35, 109228. 10.1016/j.celrep.2021.109228 34107254 PMC8206957

[B45] Huang Y. Cheng L. Turchinovich A. Mahairaki V. Troncoso J. C. Pletniková O. (2020). Influence of Species and Processing Parameters on Recovery and Content of Brain Tissue-Derived Extracellular Vesicles. J. Extracell Vesicles 9, 1785746. 10.1080/20013078.2020.1785746 32944174 PMC7480582

[B46] Hussain R. Z. Miller-Little W. A. Doelger R. Cutter G. R. Loof N. Cravens P. D. (2018). Defining Standard Enzymatic Dissociation Methods for Individual Brains and Spinal Cords in EAE. Neurol. Neuroimmunol Neuroinflamm 5, e437. 10.1212/NXI.0000000000000437 29359175 PMC5773844

[B47] Joshi P. Turola E. Ruiz A. Bergami A. Libera D. D. Benussi L. (2014). Microglia Convert Aggregated Amyloid-β into Neurotoxic Forms through the Shedding of Microvesicles. Cell Death Differ 21, 582–593. 10.1038/cdd.2013.180 24336048 PMC3950321

[B48] Karasu E. Eisenhardt S. U. Harant J. Huber-Lang M. (2018). Extracellular Vesicles: Packages Sent With Complement. Front Immunol 9, 721. 10.3389/fimmu.2018.00721 29696020 PMC5904200

[B49] Keren-Shaul H. Spinrad A. Weiner A. Matcovitch-Natan O. Dvir-Szternfeld R. Ulland T. K. (2017). A Unique Microglia Type Associated with Restricting Development of Alzheimer's Disease. Cell 169, 1276–e17. 10.1016/j.cell.2017.05.018 28602351

[B50] Kokubo H. Saido T. C. Iwata N. Helms J. B. Shinohara R. Yamaguchi H. (2005). Part of Membrane-Bound Abeta Exists in Rafts within Senile Plaques in Tg2576 Mouse Brain. Neurobiol. Aging 26, 409–418. 10.1016/j.neurobiolaging.2004.04.008 15653169

[B51] Lee K. Kim H. An K. Kwon O. B. Park S. Cha J. H. (2016). Replenishment of microRNA-188-5p Restores the Synaptic and Cognitive Deficits in 5XFAD Mouse Model of Alzheimer's Disease. Sci. Rep. 6, 34433. 10.1038/srep34433 27708404 PMC5052619

[B52] Liu J. Wang L. Harvey-White J. Osei-Hyiaman D. Razdan R. Gong Q. (2006). A Biosynthetic Pathway for Anandamide. Proc. Natl. Acad. Sci. U S A. 103, 13345–13350. 10.1073/pnas.0601832103 16938887 PMC1557387

[B53] Liu P. Reichl J. H. Rao E. R. McNellis B. M. Huang E. S. Hemmy L. S. (2017). Quantitative Comparison of Dense-Core Amyloid Plaque Accumulation in Amyloid-β Protein Precursor Transgenic Mice. J. Alzheimers Dis. 56, 743–761. 10.3233/JAD-161027 28059792 PMC5272806

[B54] Lizarbe M. A. Barrasa J. I. Olmo N. Gavilanes F. Turnay J. (2013). Annexin-phospholipid Interactions. Functional Implications. Int. J. Mol. Sci. 14, 2652–2683. 10.3390/ijms14022652 23358253 PMC3588008

[B55] Mahaman Y. A. R. Huang F. Kessete Afewerky H. Maibouge T. M. S. Ghose B. Wang X. (2019). Involvement of Calpain in the Neuropathogenesis of Alzheimer's Disease. Med. Res. Rev. 39, 608–630. 10.1002/med.21534 30260518 PMC6585958

[B56] Maphis N. Xu G. Kokiko-Cochran O. N. Jiang S. Cardona A. Ransohoff R. M. (2015). Reactive Microglia Drive Tau Pathology and Contribute to the Spreading of Pathological Tau in the Brain. Brain 138, 1738–1755. 10.1093/brain/awv081 25833819 PMC4542622

[B57] Mathys H. Davila-Velderrain J. Peng Z. Gao F. Mohammadi S. Young J. Z. (2019). Single-cell Transcriptomic Analysis of Alzheimer's Disease. Nature 570, 332–337. 10.1038/s41586-019-1195-2 31042697 PMC6865822

[B58] Matsuda S. Giliberto L. Matsuda Y. McGowan E. M. D'Adamio L. (2008). BRI2 Inhibits Amyloid Beta-Peptide Precursor Protein Processing by Interfering with the Docking of Secretases to the Substrate. J. Neurosci. 28, 8668–8676. 10.1523/JNEUROSCI.2094-08.2008 18753367 PMC3844774

[B59] Mehta D. Jackson R. Paul G. Shi J. Sabbagh M. (2017). Why Do Trials for Alzheimer's Disease Drugs Keep Failing? A Discontinued Drug Perspective for 2010-2015. Expert Opin. Investig. Drugs 26, 735–739. 10.1080/13543784.2017.1323868 PMC5576861 28460541

[B60] Mellott T. J. Pender S. M. Burke R. M. Langley E. A. Blusztajn J. K. (2014). IGF2 Ameliorates Amyloidosis, Increases Cholinergic Marker Expression and Raises BMP9 and Neurotrophin Levels in the hippocampus of the APPswePS1dE9 Alzheimer's Disease Model Mice. PLoS One 9, e94287. 10.1371/journal.pone.0094287 24732467 PMC3986048

[B61] Merrill A. H. Jr. (2011). Sphingolipid and Glycosphingolipid Metabolic Pathways in the Era of Sphingolipidomics. Chem. Rev. 111, 6387–6422. 10.1021/cr2002917 21942574 PMC3191729

[B62] Miltenberger-Miltenyi G. Cruz-Machado A. R. Saville J. Conceição V. A. Calado Â. Lopes I. (2020). Increased Monohexosylceramide Levels in the Serum of Established Rheumatoid Arthritis Patients. Rheumatology (Oxford) 59, 2085–2089. 10.1093/rheumatology/kez545 31808525

[B63] Miranda A. M. Lasiecka Z. M. Xu Y. Neufeld J. Shahriar S. Simoes S. (2018). Neuronal Lysosomal Dysfunction Releases Exosomes Harboring APP C-Terminal Fragments and Unique Lipid Signatures. Nat. Commun. 9, 291. 10.1038/s41467-017-02533-w 29348617 PMC5773483

[B64] Muraoka S. DeLeo A. M. Sethi M. K. Yukawa-Takamatsu K. Yang Z. Ko J. (2020). Proteomic and Biological Profiling of Extracellular Vesicles from Alzheimer's Disease Human Brain Tissues. Alzheimers Dement 16, 896–907. 10.1002/alz.12089 32301581 PMC7293582

[B65] Muraoka S. Jedrychowski M. P. Iwahara N. Abdullah M. Onos K. D. Keezer K. J. (2021). Enrichment of Neurodegenerative Microglia Signature in Brain-Derived Extracellular Vesicles Isolated from Alzheimer's Disease Mouse Models. J. Proteome Res. 20, 1733–1743. 10.1021/acs.jproteome.0c00934 33534581 PMC7944570

[B66] Niimura Y. Moue T. Takahashi N. Nagai K. (2010). Modification of Sphingoglycolipids and Sulfolipids in Kidney Cell Lines under Heat Stress: Activation of Monohexosylceramide Synthesis as a Ceramide Scavenger. Glycobiology 20, 710–717. 10.1093/glycob/cwq018 20157020

[B67] Nugent A. A. Lin K. van Lengerich B. Lianoglou S. Przybyla L. Davis S. S. (2020). TREM2 Regulates Microglial Cholesterol Metabolism upon Chronic Phagocytic Challenge. Neuron 105, 837–e9. 10.1016/j.neuron.2019.12.007 31902528

[B68] Orre M. Kamphuis W. Osborn L. M. Jansen A. H. P. Kooijman L. Bossers K. (2014). Isolation of Glia from Alzheimer's Mice Reveals Inflammation and Dysfunction. Neurobiol. Aging 35, 2746–2760. 10.1016/j.neurobiolaging.2014.06.004 25002035

[B69] Pascual M. Ibáñez F. Guerri C. (2020). Exosomes as Mediators of Neuron-Glia Communication in Neuroinflammation. Neural Regen. Res. 15, 796–801. 10.4103/1673-5374.268893 31719239 PMC6990780

[B70] Pascual-Lucas M. Viana da Silva S. Di Scala M. Garcia-Barroso C. González-Aseguinolaza G. Mulle C. (2014). Insulin-like Growth Factor 2 Reverses Memory and Synaptic Deficits in APP Transgenic Mice. EMBO Mol. Med. 6, 1246–1262. 10.15252/emmm.201404228 25100745 PMC4287930

[B71] Peng K. Y. Pérez-González R. Alldred M. J. Goulbourne C. N. Morales-Corraliza J. Saito M. (2019). Apolipoprotein E4 Genotype Compromises Brain Exosome Production. Brain 142, 163–175. 10.1093/brain/awy289 30496349 PMC6308312

[B72] Pérez-González R. Gauthier S. A. Kumar A. Saito M. Saito M. Levy E. (2017). A Method for Isolation of Extracellular Vesicles and Characterization of Exosomes from Brain Extracellular Space. Methods Mol. Biol. 1545, 139–151. 10.1007/978-1-4939-6728-5_10 27943212

[B73] Rajendran L. Honsho M. Zahn T. R. Keller P. Geiger K. D. Verkade P. (2006). Alzheimer's Disease Beta-Amyloid Peptides Are Released in Association with Exosomes. Proc. Natl. Acad. Sci. U S A. 103, 11172–11177. 10.1073/pnas.0603838103 16837572 PMC1544060

[B74] Rappsilber J. Mann M. Ishihama Y. (2007). Protocol for Micro-purification, Enrichment, Pre-fractionation and Storage of Peptides for Proteomics Using StageTips. Nat. Protoc. 2, 1896–1906. 10.1038/nprot.2007.261 17703201

[B75] Sanchez-Mejia R. O. Newman J. W. Toh S. Yu G. Q. Zhou Y. Halabisky B. (2008). Phospholipase A2 Reduction Ameliorates Cognitive Deficits in a Mouse Model of Alzheimer's Disease. Nat. Neurosci. 11, 1311–1318. 10.1038/nn.2213 18931664 PMC2597064

[B76] Sardar Sinha M. Ansell-Schultz A. Civitelli L. Hildesjö C. Larsson M. Lannfelt L. (2018). Alzheimer's Disease Pathology Propagation by Exosomes Containing Toxic Amyloid-Beta Oligomers. Acta Neuropathol. 136, 41–56. 10.1007/s00401-018-1868-1 29934873 PMC6015111

[B77] Schartz N. D. Tenner A. J. (2020). The Good, the Bad, and the Opportunities of the Complement System in Neurodegenerative Disease. J. Neuroinflammation 17, 354. 10.1186/s12974-020-02024-8 33239010 PMC7690210

[B78] Serrano-Pozo A. Frosch M. P. Masliah E. Hyman B. T. (2011). Neuropathological Alterations in Alzheimer Disease. Cold Spring Harb Perspect. Med. 1, a006189. 10.1101/cshperspect.a006189 22229116 PMC3234452

[B79] Showalter M. R. Berg A. L. Nagourney A. Heil H. Carraway K. L. 3rd Fiehn O. (2020). The Emerging and Diverse Roles of Bis(monoacylglycero) Phosphate Lipids in Cellular Physiology and Disease. Int. J. Mol. Sci. 21. 10.3390/ijms21218067 PMC7663174 33137979

[B80] Simon A. (2014). Cholesterol Metabolism and Immunity. N. Engl. J. Med. 371, 1933–1935. 10.1056/NEJMcibr1412016 25390746

[B81] Spann N. J. Glass C. K. (2013). Sterols and Oxysterols in Immune Cell Function. Nat. Immunol. 14, 893–900. 10.1038/ni.2681 23959186

[B82] Srinivasan K. Friedman B. A. Etxeberria A. Huntley M. A. van der Brug M. P. Foreman O. (2020). Alzheimer's Patient Microglia Exhibit Enhanced Aging and Unique Transcriptional Activation. Cel Rep 31, 107843. 10.1016/j.celrep.2020.107843 PMC7422733 32610143

[B83] Su H. Rustam Y. H. Masters C. L. Makalic E. McLean C. A. Hill A. F. (2021). Characterization of Brain-Derived Extracellular Vesicle Lipids in Alzheimer's Disease. J. Extracell Vesicles 10, e12089. 10.1002/jev2.12089 34012516 PMC8111496

[B84] Su W. Aloi M. S. Garden G. A. (2016). MicroRNAs Mediating CNS Inflammation: Small Regulators with Powerful Potential. Brain Behav. Immun. 52, 1–8. 10.1016/j.bbi.2015.07.003 26148445 PMC5030842

[B85] Szklarczyk D. Gable A. L. Lyon D. Junge A. Wyder S. Huerta-Cepas J. (2019). STRING v11: Protein-Protein Association Networks With Increased Coverage, Supporting Functional Discovery in Genome-Wide Experimental Datasets. Nucleic Acids Res. 47, D607–D13. 10.1093/nar/gky1131 30476243 PMC6323986

[B86] Takatori S. Wang W. Iguchi A. Tomita T. (2019). Genetic Risk Factors for Alzheimer Disease: Emerging Roles of Microglia in Disease Pathomechanisms. Adv. Exp. Med. Biol. 1118, 83–116. 10.1007/978-3-030-05542-4_5 30747419

[B87] Tang S.-Y. Liu D.-X. Li Y. Wang K.-J. Wang X.-F. Su Z.-K. (2020). Caspr1 Facilitates sAPPα Production by Regulating α-Secretase ADAM9 in Brain Endothelial Cells. Front. Mol. Neurosci. 13, 23. 10.3389/fnmol.2020.00023 32210761 PMC7068801

[B88] Tenner A. J. (2020). Complement-Mediated Events in Alzheimer's Disease: Mechanisms and Potential Therapeutic Targets. J. Immunol. 204, 306–315. 10.4049/jimmunol.1901068 31907273 PMC6951444

[B89] Théry C. Witwer K. W. Aikawa E. Alcaraz M. J. Anderson J. D. Andriantsitohaina R. (2018). Minimal Information for Studies of Extracellular Vesicles 2018 (MISEV2018): a Position Statement of the International Society for Extracellular Vesicles and Update of the MISEV2014 Guidelines. J. Extracell Vesicles 7, 1535750. 10.1080/20013078.2018.1535750 30637094 PMC6322352

[B90] Thion M. S. Garel S. (2018). Microglia Under the Spotlight: Activity and Complement-Dependent Engulfment of Synapses. Trends Neurosci 41, 332–34. 10.1016/j.tins.2018.03.017 29801526

[B91] Vassileff N. Vella L. J. Rajapaksha H. Shambrook M. Kenari A. N. McLean C. (2020). Revealing the Proteome of Motor Cortex Derived Extracellular Vesicles Isolated from Amyotrophic Lateral Sclerosis Human Postmortem Tissues. Cells 9, 1709. 10.3390/cells9071709 PMC7407138 32708779

[B92] Vella L. J. Scicluna B. J. Cheng L. Bawden E. G. Masters C. L. Ang C. S. (2017). A Rigorous Method to Enrich for Exosomes from Brain Tissue. J. Extracell Vesicles 6, 1348885. 10.1080/20013078.2017.1348885 28804598 PMC5533148

[B93] Wang B. Wu L. Chen J. Dong L. Chen C. Wen Z. (2021). Metabolism Pathways of Arachidonic Acids: Mechanisms and Potential Therapeutic Targets. Signal. Transduct Target. Ther. 6, 94. 10.1038/s41392-020-00443-w 33637672 PMC7910446

[B94] Wang G. Dinkins M. He Q. Zhu G. Poirier C. Campbell A. (2012). Astrocytes Secrete Exosomes Enriched with Proapoptotic Ceramide and Prostate Apoptosis Response 4 (PAR-4): Potential Mechanism of Apoptosis Induction in Alzheimer Disease (AD). J. Biol. Chem. 287, 21384–21395. 10.1074/jbc.M112.340513 22532571 PMC3375560

[B95] Willis C. M. Ménoret A. Jellison E. R. Nicaise A. M. Vella A. T. Crocker S. J. (2017). A Refined Bead-free Method to Identify Astrocytic Exosomes in Primary Glial Cultures and Blood Plasma. Front. Neurosci. 11, 335. 10.3389/fnins.2017.00335 28663721 PMC5471332

[B96] Winston C. N. Goetzl E. J. Akers J. C. Carter B. S. Rockenstein E. M. Galasko D. (2016). Prediction of Conversion from Mild Cognitive Impairment to Dementia with Neuronally Derived Blood Exosome Protein Profile. Alzheimers Dement (Amst) 3, 63–72. 10.1016/j.dadm.2016.04.001 27408937 PMC4925777

[B97] Winston C. N. Romero H. K. Ellisman M. Nauss S. Julovich D. A. Conger T. (2019). Assessing Neuronal and Astrocyte Derived Exosomes from Individuals with Mild Traumatic Brain Injury for Markers of Neurodegeneration and Cytotoxic Activity. Front. Neurosci. 13, 1005. 10.3389/fnins.2019.01005 31680797 PMC6797846

[B98] Wong M. Y. Lewis M. Doherty J. J. Shi Y. Cashikar A. G. Amelianchik A. (2020). 25-Hydroxycholesterol Amplifies Microglial IL-1β Production in an apoE Isoform-dependent Manner. J. Neuroinflammation 17, 192. 10.1186/s12974-020-01869-3 32552741 PMC7298825

[B99] Xue F. Du H. (2021). TREM2 Mediates Microglial Anti-inflammatory Activations in Alzheimer's Disease: Lessons Learned from Transcriptomics. Cells 10, 321. 10.3390/cells10020321 33557250 PMC7913972

[B100] Yan Z. Zhou Z. Wu Q. Chen Z. B. Koo E. H. Zhong S. (2020). Presymptomatic Increase of an Extracellular RNA in Blood Plasma Associates with the Development of Alzheimer's Disease. Curr. Biol. 30, 1771–e3. 10.1016/j.cub.2020.02.084 32220323

[B101] Yiannopoulou K. G. Anastasiou A. I. Zachariou V. Pelidou S. H. (2019). Reasons for Failed Trials of Disease-Modifying Treatments for Alzheimer Disease and Their Contribution in Recent Research. Biomedicines 7, 97. 10.3390/biomedicines7040097 PMC6966425 31835422

[B102] Yilmaz M. Yalcin E. Presumey J. Aw E. Ma M. Whelan C. W. (2021). Overexpression of Schizophrenia Susceptibility Factor Human Complement C4A Promotes Excessive Synaptic Loss and Behavioral Changes in Mice. Nat Neurosci, 24, 214–224. 10.1038/s41593-020-00763-8 33353966 PMC8086435

[B103] Yuyama K. Sun H. Sakai S. Mitsutake S. Okada M. Tahara H. (2014). Decreased Amyloid-β Pathologies by Intracerebral Loading of Glycosphingolipid-Enriched Exosomes in Alzheimer Model Mice. J. Biol. Chem. 289, 24488–24498. 10.1074/jbc.M114.577213 25037226 PMC4148874

[B104] Zhang L. Wang J. Wang J. Yang B. He Q. Weng Q. (2020). Role of DJ-1 in Immune and Inflammatory Diseases. Front. Immunol. 11, 994. 10.3389/fimmu.2020.00994 32612601 PMC7308417

[B105] Zhu C. Bilousova T. Focht S. Jun M. Elias C. J. Melnik M. (2021). Pharmacological Inhibition of nSMase2 Reduces Brain Exosome Release and α-synuclein Pathology in a Parkinson's Disease Model. Mol. Brain 14, 70. 10.1186/s13041-021-00776-9 33875010 PMC8056538

